# Genetic Diversity of *Colletotrichum* spp. an Endophytic Fungi in a Medicinal Plant, Brazilian Pepper Tree

**DOI:** 10.5402/2012/215716

**Published:** 2012-05-30

**Authors:** J. S. Lima, J. G. Figueiredo, R. G. Gomes, D. Stringari, E. H. Goulin, D. Adamoski, V. Kava-Cordeiro, L. V. Galli-Terasawa, C. Glienke

**Affiliations:** Department of Genetics, Centro Politécnico, Universidade Federal do Paraná (UFPR), Box 19071, 81531-990 Curitiba, PR, Brazil

## Abstract

In this study, we reported thirty-nine endophytic fungi identified as *Colletotrichum* spp. associated with Brazilian pepper tree or aroeira (*Schinus terebinthifolius* Raddi. Anacardiaceae) in Paraná state, Brazil. These endophytes were identified by morphological and molecular methods, using PCR taxon-specific with *Ca*Int/ITS4, *Cg*Int/ITS4, and *Col*1/ITS4 primers, which amplify specific bands in *C. acutatum*, *C. gloeosporioides lato sensu,* and *Colletotrichum boninensis*, respectively, and by DNA sequence analysis of the nrDNA internal transcribed spacer region (ITS1, 5.8S, ITS2). We also assayed the presence of dsRNA particles in *Colletotrichum* spp. isolates. Combining both morphological characters and molecular data, we identified the species *C. gloeosporioides*, *C. boninense,* and *C. simmondsii*. However, we found a high genetic variability intraspecific in *C. gloeosporioides* which suggests the existence of several other species. Bands of double-stranded RNA (dsRNA) were detected in three of thirty-nine isolates. Identity of these bands was confirmed by RNAse, DNAse, and S1 nuclease treatments for the isolates LGMF633, LGMF726, and LGMF729. This is the first study reporting these particles of dsRNA in *C. gloeosporioides*.

## 1. Introduction

Potential sources of new natural products have been explored in medicine, agriculture, and industry. Endophytic fungi have been recognized as useful sources of bioactive secondary metabolites [1], especially those isolated from medicinal plants [2, 3]. Various important characteristics are currently attributed, such as the increase in resistance to stressful conditions; alteration in physiological properties; production of phytohormones, toxins, medicinal substances, immunosuppressants, antitumor agents, and compounds of biotechnological interest such as enzymes [1, 4–13].

Brazilian pepper tree also called aroeira (*Schinus terebinthifolius *Raddi-Anacardiaceae) is native to Argentina, Brazil, and Paraguay [14]. In Brazil, the bark leaves and fruits have been used in popular medicine due to their medicinal properties [15–18]. Actions anti-inflammatory and antiseptic for treatment of wounds, urinary, and respiratory infections are listed as medicinal properties popularly known [19]. Studies showed antimicrobial activity [15, 18, 20–24], antifungal activity [25, 26], as antioxidant [15, 27], and antitumor [18, 28]. Despite its importance, there are a few records about the endophytic community in this plant.

*Colletotrichum* has been isolated from numerous plant species especially as symptomatic pathogens but can be found as asymptomatic endophytes. The genus has wide geographic distribution, being more important in the tropics. Studies involving the complex *C. gloeosporioides *and *C. boninense* revealed high genetic variability and molecular diversity [21, 29–31]. There is significant interest in developing a fast, simple, and efficient method to identify species of *Colletotrichum*. Several authors have described new species and morphological characteristics associated to species of *Colletotrichum* [32–36]. Afanador-Kafuri et al. [32] developed specific primers to *C. boninense *[34]. The species *C. acutatum* was also organized and divided into three species, *C. acutatum*, *C. fioriniae *comb. *et* stat. nov., and *C. simmondsii *sp. nov. [36].

Several authors have investigated the influence of viral particles on fungi [37]. Fungal virus genomes are commonly composed of dsRNA that can modulate plant-fungal symbioses [38]. The associations between fungal viruses and their hosts are similar to those involved in plant-endophyte interactions [37]. Changes in morphological characteristics and increased production of conidia have been reported as associated with the presence of dsRNA in *Beauveria bassiana *[39], *Metarhizium anisopliae* [40, 41], and *Nectria radicicola* [34].

In the present study, we isolated endophytic fungi from leaves of medicinal tree called aroeira (*Schinus terebinthifolius *Raddi). These endophytes were identified by morphological and molecular methods. We also assayed the presence of dsRNA particles in *Colletotrichum* spp. isolates.

## 2. Material and Methods

### 2.1. Fungal Isolates

Isolates were obtained from leaves of plants of Brazilian pepper tree (*S. terebinthifolius *Raddi), located in the campus of the University of Paraná, Paraná, Brazil. The isolates were obtained as described by Petrini [42] and identified by macroscopic and microscopic reproductive structures after growth on PDA medium. The cultures are permanently stored in the fungal collection of the Laboratory of Microorganisms (LabGeM-UFPR), Paraná, Brazil.

## 3. Molecular Characterization

### 3.1. DNA Extraction

*Colletotrichum* isolates were grown on PDA medium for 3 days at 28°C. The mycelium was harvested, lyophilized for 24 h, and ground with a mortar and pestle under liquid nitrogen. Genomic DNA was obtained according to methods described by Raeder and Broda [43], modified by Glienke-Blanco et al. [44].

### 3.2. Species-Specific PCR

Species-specific amplifications were performed using the primer ITS4 [45] with specific primers for *C. gloeosporioides *complex (*Cg*Int: 5′-GGCCTCCCGCCTCCGGGCGG-3′) [46], *C. boninense* (*Col*1: 5′-GCCGTCCCCTGAAAAG) [47], or *C. acutatum *complex (CaInt2: 5′-GGGGAAGCCTCTCGCGG-3′) [32].

According to the method described by Pileggi et al. [47], PCR reactions were performed in a total volume of 25 *μ*L, containing 1X buffer solution, 1.5 mM MgCl_2_, 0.2 mM of each dNTP (Invitrogen, CA, USA), 0.5 *μ*M primer, 1.5 Unit of *Taq* DNA polymerase (Invitrogen, CA, USA), and 20 ng of genomic DNA. Amplifications were carried using the following conditions: an initial denaturation at 95°C for 5 min, followed by 40 cycles of 30 s at 95°C, 30 s at 65°C, and 1.5 min at 72°C, and a final extension at 72°C for 3 min.

To identify *C. acutatum* and *C. gloeosporioides* complex, the PCR reactions were performed as previously described. Amplifications were carried out in a gradient thermocycler with an initial denaturation period of 5 min at 95°C, followed by one cycle of 30 sec at 94°C, 45 seconds at 62°C, 90 seconds at 72°C, one cycle of 30 seconds at 94°C, 45 seconds at 60°C, 90 seconds at 72°C, followed by 33 cycles of 30 seconds at 94°C, 45 seconds at 58°C e 90 seconds at 72°C, and a final extension period of 3 minutes at 72°C.

Genomic DNA of positive control of *C. gloeosporioides* (Col11), *C. boninense* (Col7), is obtained from Pileggi et al. [47]. Genomic DNAs of *C. acutatum* strains FDC89A08 and FDC31A08 were obtained from Fundecitrus, SP.

### 3.3. DNA Analysis and Sequencing

The primers V9G [48] and ITS4 [45] were used to amplify the internal transcribed spacer region (ITS) of the nuclear ribosomal RNA operon, including the 3′ end of the 18S rRNA, the first internal transcribed spacer region, the 5.8S rRNA gene; the second internal transcribed spacer region and the 5′ end of the 28S rRNA gene. PCR was performed in total reaction volume of 50 *μ*L, which was composed of 1 × PCR  Buffer (Applied Biosystems, Foster City, USA), 2 mM MgCl2, 40 *μ*M dNTPs, 0.08 *μ*M of each forward and reverse primer, 0.5 U of *Taq *DNA polymerase (Roche Diagnostics, Indianapolis, USA), and 1–10 ng of genomic DNA. Thirty cycles were performed: 94°C for 30 s, 56°C for 1 min, 72°C for 1 min, and 2 min initial and terminal delay. The second condition had a total reaction volume of 12.5 *μ*L, which was composed of 1 × PCR  Buffer (Bioline GmbH, Luckenwalde, Germany), 5.6% DMSO (v/v), 2 mM MgCl_2_, 20 *μ*M dNTPs, 0.2 *μ*M of each forward and reverse primer, 0.25 U of BioTaq *Taq *DNA polymerase (Bioline GmbH, Luckenwalde, Germany), and 1–10 ng of genomic DNA. The PCR cycle conditions were 5 min of 94°C, followed by 40 cycles of 94°C for 30 s, 52°C for 30 s, 72°C for 30 s, and a final elongation step at 72°C for 7 min.

Amplified rDNA fragments were cleaned with 50 *μ*L 20% PEG and resuspended in 15 *μ*L of ultrapure water. To confirm the presence of DNA in the sample, 1 *μ*L was applied on a 1.4% agarose gel. rDNA Internal Transcribed Spacer (ITS) was sequenced with primers ITS4 and ITS1 [45]. PCR was performed in 10 *μ*L volumes of a reaction mixture containing sterile distilled water, 0.5 *μ*L PCR buffer (10x, Applied Biosystems), 0.5 *μ*L of primer (50 pmol), 0.5 *μ*L of Big Dye (Applied Biosystems), and 1 *μ*L PCR products. Thirty five cycles were performed: 96°C for 10 s (denaturation), 50°C for 5 s (annealing), 60°C for 4 min (extension), and 60 s initial and terminal delay. Sequencing was performed on an ABI 3130 automatic sequencer (*Perkin-Elmer*, Massachusetts, USA).

### 3.4. Sequence Assembly and Alignment

Sequences were edited using BioEdit 7.0 [40]. ITS sequences were aligned on the basis of similarity by means of the sequence editor CLUSTAL-W 1.7 [49]. Sequence analysis was performed using the sequence alignment software BLASTn run against the NCBI database (National Center for Biotechnology Information website (http://www.ncbi.nlm.nih.gov/)).

Maximum likelihood tree search was done with GARLI version 2.0 [50]. The algorithm settings was the default, with 1.000 bootstraps (50 runs of 20 repetitions). Bayesian tree search was done with parallel MrBayes version 3.1.2 [51, 52], using 20.000.000 generations and 4 independents runs. The Model, SYM+I, was selected with jMODELTEST v0.1.1 [53] using Bayesian Information Criterion (BIC). The traces were analysed with Tracer v1.5 [54] and AWTY [55] to evaluate the stationary phase, setting the burn-in to 2.000.000 generations. To merge bootstrap replicates and posterior probabilities and summarize the consensus tree, SumTrees [36] from DendroPy package version 3.7.0 [23] was used.

### 3.5. dsRNA Analysis

After genomic DNA extraction was performed electrophoresis on 0.7% agarose gel to observe the occurrence of bands of dsRNA. For confirmation, the total DNA of the isolates was submitted separately to the treatment of enzymatic digestion with RNAse, DNAse, and S1 Nuclease. Three digestions were performed as described by Azevedo et al. [1].

## 4. Results

### 4.1. Species-Specific PCR

The isolates were investigated in PCR with specific primers for *C. acutatum, C. gloeosporioides, *and *C. boninense* (Table 1). Isolates LGMF625, 666, 667, and 738 did not amplify the expected band with *Cg*Int/ITS4 primers. LGMF666 showed amplification using primer *Col1*/ITS4, that amplifies a specific band for *C. boninense* and LGMF738 amplifies a specific band with *Ca*Int/ITS4 primer, and was identified as* C. acutatum* complex (Table 1).

### 4.2. DNA Analysis and Sequencing

**Figure 1:**
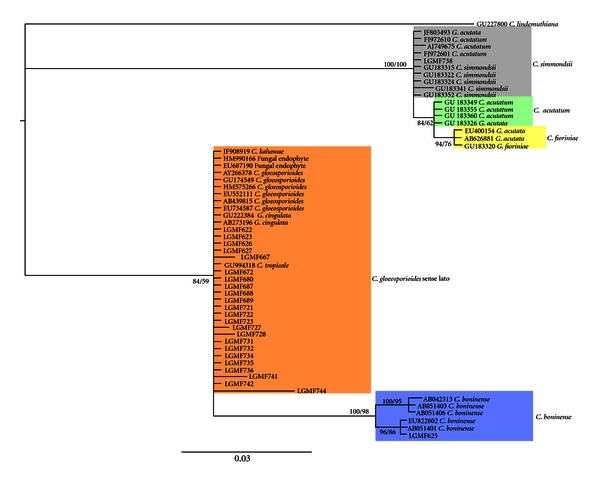
Phylogenetic relationships among the studied strains based on maximum likelihood and Bayesian inference. Values below branches represent either bootstrap support values (maximum likelihood tree) or posterior probabilities (Bayesian inference).

Twenty five isolates were sequenced for the ITS1-5.8S-ITS2 of rDNA, generating fragments between 600 and 700 bp. Phylogenetic analysis grouped the *Colletotrichum *isolates into three clades (Figure 1). The first clade included the *C. acutatum *complex species, with 100% bootstrap support. The isolate LGMF738 clustered with *C. simmondsii *(GU183331) holotype strain. All *Colletotrichum *isolates that clustered in clade II included isolates of the *C. gloeosporioides *from GenBank (AB439815; AB273196; EU734587; EU552111; EU687190) with 84% bootstrap support. The third clade clustered the isolate LGMF625 with *C*. *boninense* species (EU822802 and AB051401).

### 4.3. dsRNA Analyses

After electrophoresis of genomic DNA was observed bands of approximately 3000 bp indicating the presence of dsRNA in six isolates of *C. gloeosporioides *complex LGMF633, 638, 689, 726, 729, and LGMF736 (Table 1).

## 5. Discussion

The taxonomy of *Colletotrichum* is confused, both for the anamorphic species and its teleomorph *Glomerella*. The combined use of molecular diagnostic tools along with traditional morphological techniques is at present an appropriate approach for studying *Colletotrichum* species complexes [6, 47].

Afanador-Kafuri et al. [32] proposed the use of two pairs of primers for the identification of *Colletotrichum *species, one for *C. gloeosporioides *(CgInt) and another for *Colletotrichum* sp (Col1). Moriwaki et al. [30] proposed the classification of isolates originally identified as *C. gloeosporioides* as belonging to a new species, they called *C. boninense*. Pileggi et al. [47] suggested that the primers pair developed by Afanador-Kafuri et al. [32] for *Colletotrichum* sp amplify isolates of the new species *C. boninense* proposed by Moriwaki et al. [30]. Moriwaki et al. [30] showed that the ITS1 region of *C*. *boninense *was 190 bp, whereas, for *C*. *gloeosporioides, *this region was 171 bp. Consequently, the difference between *C*. *boninense *and *C*. *gloeosporioides *should reflect interspecific relationships and should be further investigated.

In this paper, we proposed PCR identification species-specific anticipated results, enabling identification of approximately 70% of isolates without the need for extensive morphological analysis. ITS sequence analysis confirmed species-specific results and resolved the identification of *C. boninense *(LGMF625) and *C. gloeosporioides *complex. However, the PCR species-specific mistakenly identified the isolate LGMF738 as *C. acutatum* when in fact it belongs to the new species *C. simmondsii* (Table 1).

The analyzed Brazilian pepper trees were colonized by three different species of *Colletotrichum* and showed high genetic diversity; including the species *C. gloeosporioides sense lato*, *C. boninense, *and *C. simmondsii*. The ecological roles of endophytes are diverse and varied. *Colletotrichum gloeosporioides *complex is a worldwide plant pathogen that infects many plant species. These isolates will need more examination to ensure the correct identification. Zou et al. [27] reported one endophytic isolate of *C. gloeosporioides* from stem of *Artemisia mongolica* that produced the colletotric acid, with antimicrobial activity against *Bacillus subtilis*, *Staphylococcus aureus*, *Sarcina lutea,* and *Helminthosporium sativum* [27].

Many studies discriminate *Colletotrichum* complex using ribosomal ITS sequence data; however, due to the limited number of informative sites been identified, other regions of the Genome, such as the *β*-tubulin gene, have been identified suitable for the phylogenetic reconstruction [56]. Hyde et al. [57] suggest epitypification and use of multilocus phylogeny to delimit species and gain a better understanding of the genus. Our data corroborated the existence of more than one species in the *C. gloeosporioides *complex. Also, our data corroborated the reassessment of *Colletotrichum acutatum *complex and the new species *C. simmondsii* introduced by Shivas and Tan [58]. It was the first report of *C. simmondsii *as an endophyte from *Schinus terebinthifolius*. The host range and host specificity of *C. simmondsii* are not clear [36].

This paper is the first study describing the existence of dsRNA particles in *C. gloeosporioides* isolates. The presence of dsRNA in entomopathogenic fungi is described in a long time [41, 59–63]. Dalzoto et al. [39] described the horizontal transfer and hypovirulence associated with dsRNA in the fungus *B. bassiana*. The authors suggest the increased production of conidia in strains without dsRNA when compared with the strains positive to dsRNA particles.

Morphological changes of colonies associated with the presence of the dsRNA particles were described in* Chalara elegans* [64], *Metarhizium anisopliae* [40, 41], *Diaporthe ambigua* [62], and *Nectria radicicola* [34].

Double-stranded RNA viruses have been described for a long time in a wide variety of filamentous fungi and yeasts [9, 37, 65–67]. Marquez et al. [38] suggested the associations between fungal viruses and their hosts are similar to plant-endophyte associations. In this study, Marquez et al. [38] found no differences in colony morphology among isolates with dsRNA and those free dsRNA. Also, the authors did not find any association between the presences of dsRNA and genetically different groups. In the *Colletotrichum* genus, it is not yet known the influence that these particles can have on fungi morphology or physiology. So, we suggest the investigation by scanning electron microscopy and also by the study of these strains after cure (elimination) of dsRNA.

## Figures and Tables

**Table 1:** Identification of *Colletotrichum *using primers taxon-specific CaInt-ITS4, CgInt-ITS4 and Col-ITS4.

Species	Strain number	PCR identification			ITS
		CaInt-ITS4	CgInt-ITS4	Col-ITS4	
*C. boninense*	LGMF625	*−*	*−*	*−*	*C. boninense*
	LGMF666	*−*	*−*	+	na
*C. acutatum*	LGMF738	*+*	*−*	*−*	*C. acutatum*
*C. gloeosporioides*	LGMF621	*−*	*+*	*−*	na
	LGMF622	*−*	*+*	*−*	*C. gloeosporioides*
	LGMF623	*−*	*+*	*−*	*C. gloeosporioides*
	LGMF626	*−*	*+*	*−*	*C. gloeosporioides*
	LGMF627	*−*	*+*	*−*	*C. gloeosporioides*
	LGMF633*	*−*	*+*	*−*	na
	LGMF638	*−*	*+*	*−*	na
	LGMF667	*−*	*-*	*−*	*C. gloeosporioides*
	LGMF669	*−*	*+*	*−*	na
	LGMF672	*−*	*+*	*−*	*C. gloeosporioides*
	LGMF678	*−*	*+*	*−*	na
	LGMF680	*−*	*+*	*−*	*C. gloeosporioides*
	LGMF682	*−*	*+*	*−*	na
	LGMF686	*−*	*+*	*−*	na
	LGMF687	*−*	*+*	*−*	*C. gloeosporioides*
	LGMF688	*−*	*+*	*−*	*C. gloeosporioides*
	LGMF689	*−*	*+*	*−*	*C. gloeosporioides*
	LGMF721	*−*	*+*	*−*	*C. gloeosporioides*
	LGMF722	*−*	*+*	*−*	*C. gloeosporioides*
	LGMF723	*−*	*+*	*−*	*C. gloeosporioides*
	LGMF726*	*−*	*+*	*−*	na
	LGMF727	*−*	*+*	*−*	*C. gloeosporioides*
	LGMF728	*−*	*+*	*−*	*C. gloeosporioides*
	LGMF729*	*−*	*+*	*−*	na
	LGMF730	*−*	*+*	*−*	na
	LGMF731	*−*	*+*	*−*	*C. gloeosporioides*
	LGMF732	*−*	*+*	*−*	*C. gloeosporioides*
	LGMF733	*−*	*+*	*−*	na
	LGMF734	*−*	*+*	*−*	*C. gloeosporioides*
	LGMF735	*−*	*+*	*−*	*C. gloeosporioides*
	LGMF736	*−*	*+*	*−*	*C. gloeosporioides*
	LGMF740	*−*	*+*	*−*	*na*
	LGMF741	*−*	*+*	*−*	*C. gloeosporioides*
	LGMF742	*−*	*+*	*−*	*C. gloeosporioides*
	LGMF743	*−*	*+*	*−*	na
	LGMF744	*−*	*+*	*−*	*C. gloeosporioides*

(*) dsRNA confirmed after analysis digestion with RNAse, DNAse, and S1 Nuclease. na: not available.
